# Impact of phosphorus reduction combined with biofertilizer application on soil nutrients and microbial communities in arid oasis agricultural areas

**DOI:** 10.3389/fmicb.2025.1606813

**Published:** 2025-10-03

**Authors:** Yong Ling Zhang, Rang Xiao, Yun Chen Zhao, Tongfen Li, Hong Yu Cheng, Hong Juan Zhang

**Affiliations:** ^1^College of Civil Engineering, Hexi University, Zhangye, China; ^2^Research Institute of Water Resources Protection and Utilization in Hexi Corridor, Zhangye, China; ^3^College of Water Conservancy and Hydropower Engineering, Gansu Agricultural University, Lanzhou, China; ^4^College of Agricultural and Ecological Engineering, Hexi University, Zhangye, China; ^5^College of Pastoral Agriculture Science and Technology, Lanzhou University, Lanzhou, China

**Keywords:** phosphorus reduction, biofertilizers, soil available nutrients, microbial diversity, carbon-nitrogen cycling, sustainable agriculture

## Abstract

**Introduction:**

Phosphorus reduction in agriculture is crucial for sustainable soil management, yet its interactive effects with microbial fertilizers on soil nutrient dynamics and microbial communities remain poorly understood.

**Methods:**

Here, we evaluated the impacts of phosphorus reduction at four levels [0% (P100), 15% (P85), 30% (P70), and 100% (P0)] combined with two biofertilizers—*Bacillus* subtilis (BF1) and *Bacillus mucilaginosus* (BF2)—on soil available nutrients and bacterial community structure.

**Results:**

Our results demonstrated that P85 combined with BF1 significantly enhanced soil microbial diversity, while P85 combined with BF2 notably increased the levels of available phosphorus and potassium, without significant changes in microbial diversity but with a more pronounced shift in community structure. Microbial community analysis revealed that under BF1 treatment, the proportion of *Pseudomonadota*, which dominates the carbon cycle, significantly increased. Meanwhile, BF2 treatment promoted the enrichment of *Acidobacteriota* and *Planctomycetota*, both involved in carbon and nitrogen cycles. Additionally, both biofertilizers significantly increased the abundance of aerobic and biofilm-forming bacteria. Redundancy analysis (RDA) showed that nitrogen cycle-related microbiota under BF1 treatment were the primary drivers of soil nutrient changes, whereas under BF2 treatment, *Acidobacteriota*, *Chloroflexota*, and *Actinomycetota* (involved in carbonnitrogen cycling and organic matter degradation) contributed more to nutrient enhancement. In conclusion, the application of two biofertilizers with P85 can optimize soil nutrient availability and regulate microbial community structure, with BF1 being more beneficial for maintaining microbial diversity and BF2 having a superior effect on enhancing available phosphorus and potassium.

**Conclusion:**

The combined application of biofertilizers with phosphorus reduction demonstrates potential for improving soil health, thereby providing a scientific basis for promoting sustainable agricultural development.

## 1 Introduction

Global arable land accounts for 24% of the Earth’s terrestrial area (AbdelRahman, 2023). As the fundamental resource for agricultural production, cultivated land plays an irreplaceable role in ensuring food security, maintaining ecological balance, and fostering economic development (McLaughlin and Kinzelbach, 2015; Li C. et al., 2022). Beyond its agricultural production functions, cultivated land ecosystems provide critical ecological services—including carbon sequestration, oxygen release, water conservation, and biodiversity maintenance—that play a pivotal role in mitigating climate change and sustaining regional ecological balance (DeFries et al., 2004; Wang, 2022). Soil, the core of arable land productivity, has multiple aspects of sustainability, including fertility, structure, biological activity, and resistance to degradation (Song et al., 2022). In combination, these characteristics fundamentally determine both the temporal stability of farming outputs and the enduring capacity for sustained crop productivity (Sofo et al., 2022; Jiao et al., 2022).

In agricultural land management practices, scientific fertilization serves as a critical measure for enhancing soil fertility and ensuring high crop yields (Ludwig et al., 2011). Rational fertilization practices not only directly enhance crop productivity but also improve nutrient cycling and use efficiency through optimization of soil microbial community structure (Soman et al., 2017). However, excessive fertilizer use, particularly phosphorus fertilizers, not only raises agricultural production costs but also leads to phosphorus accumulation in the soil, environmental pollution, and ecological imbalances (Li et al., 2015). Studies have shown that a moderate reduction in phosphorus fertilizer use does not decrease crop yield or soil nutrient availability (Duan et al., 2023). Combining moderate phosphorus reduction with biofertilizer can reduce the risk of agricultural non-point source pollution while optimizing soil microbial community structure and improving phosphorus fertilizer efficiency (Liu et al., 2022).

In recent years, biofertilizers have gained widespread attention for their ability to improve the soil microbial environment and enhance plant nutrient absorption (Wang et al., 2020; Liu et al., 2022). Biofertilizers, through the colonization and metabolic activities of functional microorganisms, can enhance the availability of soil nutrients, suppress soil-borne pathogens, and enhance crop resistance to stress (Kapoor et al., 2024). For instance, *Bacillus mucilaginosus*, a potassium-solubilizing bacterium, is highly effective in potassium release (Basak and Biswas, 2009). Research has shown that functional microorganisms, such as nitrogen-fixing bacteria, phosphorus-solubilizing bacteria, and organic matter-degrading bacteria, can promote the effective utilization of soil nutrients, increase plant nutrient absorption efficiency, and maintain high crop yields and soil health despite reduced chemical fertilizer use (Richardson, 2001). Therefore, exploring the feasibility of reducing phosphorus fertilizer application while combining it with microbial fertilizers (biofertilizers) to maintain soil nutrient balance has become one of the key issues in current sustainable agricultural research (Duan et al., 2023; Liu et al., 2022).

Oases play a critical role in maintaining regional food security (El Janati et al., 2021). In oasis agricultural systems, water scarcity makes the efficient use of soil nutrients particularly important (Xue et al., 2015). Proper fertilization not only promotes crop growth but also optimizes the structure of soil microbial communities, thereby influencing the cycling and utilization of soil nutrients (Li et al., 2021). However, there remains a significant research gap regarding how to effectively enhance nutrient use efficiency under reduced fertilizer input while maintaining soil health and crop productivity in saline-alkaline arid oasis environments. In particular, the synergistic effects of combining specific functional microorganisms—such as *B. subtilis* and *B. mucilaginosus*—with phosphorus reduction strategies have not been thoroughly investigated under real-field drip irrigation conditions. To address this knowledge gap, we hypothesize that the co-application of these biofertilizers with moderate phosphorus reduction can not only maintain crop yield but also enhance soil microbial activity and nutrient transformation efficiency in saline-alkaline soils. To test this hypothesis, a field experiment was conducted at the water-saving experimental station in Pingyuanbao Town, Ganzhou District, Zhangye City, a representative arid oasis region. This study integrates drip irrigation and fertigation technology to systematically evaluate the colonization dynamics of functional microbes and their impact on soil nutrient cycling, thereby providing a novel practical strategy and theoretical basis for sustainable phosphorus management and ecological intensification of oasis agriculture in arid regions.

## 2 Materials and methods

### 2.1 Study site

The Experimental Station in Pingyuanbao Town, Ganzhou District, Zhangye City, is located in the central part of the Hexi Corridor, Gansu Province, China, with specific geographic coordinates of 38°32’–39°24’ N and 100°06’–100°52’ E. The average elevation is approximately 1,474 m, and the terrain is flat. It lies within the middle oasis plain area of the Heihe River Basin, a typical agricultural irrigation zone. The annual average temperature ranges from 4.1 °C to 8.3 °C, with significant diurnal temperature variation. Annual precipitation ranges from 112.3 to 354 mm, concentrated in the summer, and the evaporation rate is as high as 2,047 mm. The area enjoys 3,085 h of sunshine annually, providing abundant solar and thermal resources. The frost-free period lasts 138–179 days, making it suitable for crop growth. The predominant soil type is saline-alkaline soil, soil organic matter was 12.4 g kg^–1^, total nitrogen 0.84 g kg^–1^, total phosphorus 0.81 g kg^–1^, and total potassium 17.9 g kg^–1^.

### 2.2 Experimental design

The experimental plots were set up with dimensions of 18 m × 4 m. A plastic film cover with a width of 0.7 m was applied to the planting rows, with a plant spacing of 0.25 m and a row spacing of 0.5 m. A 1.0 m observation path was reserved between the plot groups, and a 0.6 m observation path was left between plots to prevent cross-contamination of water and fertilizers. Nine treatments were set (Table 1) with three replications, totaling 27 plots. The base fertilizer was applied as follows: 300 kg ha^–1^ of triple superphosphate (P_2_O_5_, 46%), 150 kg ha^–1^ of potassium sulfate (K_2_O, 52%), 75 kg ha^–1^ of urea (N, 46%), and 22.5 kg ha^–1^ of zinc sulfate (ZnSO_4_⋅H_2_O, 35%). Fertilizers were applied through drip irrigation: the first application at the jointing stage (2024-06-15) consisted of 225 kg ha^–1^ of soluble nitrogen fertilizer (N, 46%); the second application at the filling stage (2024-07-20) involved 300 kg ha^–1^ of soluble nitrogen fertilizer (N, 46%); and the third application at the milking stage (2024-08-10) included 225 kg ha^–1^ of soluble nitrogen fertilizer (N, 46%). All fertilizers were provided by a local fertilizer company, with treatments kept consistent across all plots. Irrigation and weed management were conducted uniformly across the experiment. The biofertilizer was applied at a rate of 75 kg⋅ha^–1^ during the maize jointing stage (mid-June) using a localized soil incorporation method: the inoculant was placed into excavated soil around plant roots and immediately covered with backfill soil. The microbial blended fertilizer and microbial inoculum were purchased from Gansu Xingshuo Biotechnology Co., Ltd. (the number of viable bacteria ≥ 200 million/g). The solid powder inoculants of *B. subtilis* and *B. mucilaginosus* were developed through a standardized process beginning with strain activation on agar slants (NA medium for *B. subtilis* and silicate medium for *B. mucilaginosus*) at 30 °C for 48 h, followed by liquid fermentation in specialized media (LB for *B. subtilis* and low-phosphate medium for *B. mucilaginosus*) with 5% inoculum under 1:1 vvm aeration at 28 °C –30 °C for 24–36 h until reaching ≥ 10^9^ CFU/mL, where *B. subtilis* fermentation was supplemented with 0.1% MnSO_4_ to enhance sporulation while *B. mucilaginosus* received 1% potassium feldspar powder to stimulate exopolysaccharide production; post-fermentation, the broth was concentrated to ≥ 10^11^ CFU/mL via disk centrifugation (8,000 rpm, 15 °C), with *B. subtilis* undergoing additional 0.5% CaCl_2_ treatment and 40 °C heat shock for 1 h to promote sporulation, after which both concentrated cultures were blended with carrier substrates (*B. subtilis*: 500 kg peat/humic acid, 200 kg wheat bran, 50 kg diatomite, 30 kg light calcium carbonate, and 5 kg trehalose; *B. mucilaginosus*: 600 kg peat/humic acid, 150 kg wheat bran, 100 kg diatomite, 30 kg light calcium carbonate, and 10 kg trehalose) in a double-helix mixer at 20 rpm for 30 min, dried via fluidized bed (45 °C inlet air, ≤ 40 °C material temperature) to ≤ 8% moisture, and milled to 80 mesh (180 μm) before vacuum packaging, yielding final products with ≥ 2 × 10^8^ CFU/g viability, ≥ 80% survival after 40 °C/14 days stability testing, ≥ 50 mg/g citric acid secretion (*B. mucilaginosus*). According to China national bio-organic fertilizer quality standard NY884-2012 (Liu et al., 2019).

**TABLE 1:** Fertilization treatment setup.

Treatments	N (N, 46%) 825 kg ha^–1^, P (P_2_O_5_, 46%) 300 kg ha^–1^, K (K_2_O,52%) 150 kg ha^–1^, ZN (ZnSO4⋅H2O, 35%) 22.5 kg ha^–1^ of zinc sulfate	Microbial biofertilizer (75 kg ha^–1^)
CK	NPK constant fertilization	No biofertilizer applied
P100 (BF1)	NPK constant fertilization	*Bacillus subtilis* (2 billion CFU)
P100 (BF2)	NPK constant fertilization	*Bacillus mucilaginosus* (2 billion CFU)
P85 (BF1)	NK constant, 15% phosphorus reduction	*Bacillus subtilis* (2 billion CFU)
P85 (BF2)	NK constant, 15% phosphorus reduction	*Bacillus mucilaginosus* (2 billion CFU)
P70 (BF1)	NK constant, 30% phosphorus reduction	*Bacillus subtilis* (2 billion CFU)
P70 (BF2)	NK constant, 30% phosphorus reduction	*Bacillus mucilaginosus* (2 billion CFU)
P0 (BF1)	NK constant, no phosphorus fertilizer	*Bacillus subtilis* (2 billion CFU)
P0 (BF2)	NK constant, no phosphorus fertilizer	*Bacillus mucilaginosus* (2 billion CFU)

### 2.3 Soil sampling and analysis

After the corn harvest, soil samples from the 0 to 20 cm plow layer were collected from each plot using a five-point sampling method. To avoid fertilization grooves and the edges of plastic film covers, soil samples were taken with a stainless steel soil auger to prevent metal contamination. Soil samples from three replicates of the same treatment were mixed in equal amounts to form biological replicates (*n* = 3). After removing visible roots and residual plastic film, the samples were sieved through a 2 mm mesh and divided into two parts: one was stored at 4 °C for microbial analysis, and the other was air-dried and ground through a 100-mesh sieve for physicochemical analysis.

Total nitrogen (TN) was determined using the Kjeldahl method, Soil samples (0.5 g) were digested with sulfuric acid-catalyst mixture (420 °C) and distilled, followed by titration with 0.01 mol/L HCl. Total phosphorus (TP) was measured by the sodium hydroxide fusion-molybdenum-antimony colorimetric method, Samples (0.2 g) were fused with NaOH (720 °C) and analyzed via molybdenum-antimony colorimetry (UV-Vis spectrophotometer, 700 nm). Total potassium (TK) was Quantified per using HF-HClO4 digestion and flame photometry (766.5 nm potassium filter). Available nutrients were measured as follows: available phosphorus (SAP) was determined by the molybdenum-antimony colorimetric method. SAP was extracted with 0.5 mol/L NaHCO_3_ (pH 8.5; modified Olsen method). After shaking (25 °C, 30 min), supernatants were analyzed by molybdenum-blue colorimetry (882 nm). Available potassium (AK) was Extracted with 1 mol/L NH_4_OAc (pH 7.0) and measured via flame photometry. Soil organic matter (SOM) was determined by K_2_Cr_2_O_4_ oxidation. Samples (0.1 g) were heated (170 °C–180 °C, 5 min) with 0.8 mol/L K_2_Cr_2_O_7_ and concentrated H_2_SO_4_, then titrated with 0.2 mol/L FeSO_4_. Organic carbon content was converted to SOM using the Bemmelen factor (1.724). The analyses of SOM, TN, TP, TK, AP, and AK were based on the standard methods as explained in a previous study (Binkley and Vitousek, 1989; Wilke, 2005).

DNA extraction and amplification: Total DNA was extracted using the PowerSoil^®^ DNA Extraction Kit, and the quality was checked by 1% agarose gel electrophoresis. The bacterial 16S rRNA gene V3–V4 region was amplified using universal primers 338F (5′-ACTCCTACGGGAGGCAGCAG-3′) and 806R (5′-GGACTACHVGGGTWTCTAAT-3′). High-throughput sequencing: Purified PCR products were sequenced on the Illumina NovaSeq platform with paired-end sequencing (2 × 250 bp). The raw data were quality controlled using Trimmomatic and clustered into OTUs at 97% similarity using USEARCH (v11). Species annotation was performed based on the Silva database (v138).

### 2.4 Statistical analysis

All physicochemical data are presented as the mean ± standard error, and one-way analysis of variance (ANOVA) was conducted to compare the effects of different phosphorus fertilizer treatments under identical microbial fertilizer conditions on soil physicochemical properties (organic matter, total nitrogen, total phosphorus, total potassium, available phosphorus, available potassium) and soil bacterial community characteristics (OTU richness, Chao1 index, Shannon-Wiener index, Pielou index), as well as compositional differences at the bacterial phylum level. Tukey’s HSD post hoc test was employed for multiple comparisons. It should be noted that during the one-way ANOVA, the same control group (CK) data were used for both microbial fertilizer treatments combined with different phosphorus fertilizer applications, which are uniformly indicated in gray in the corresponding figures. All statistical analyses described above were performed using SPSS 26.0.

Using the QIIME 2 platform, high-quality sequences were clustered into operational taxonomic units (OTUs) at a 97% similarity threshold to generate an OTU table. The table was then rarefied to an even sequencing depth to minimize the effects of varying sequencing volumes on diversity metrics. Based on the rarefied data, α-diversity was assessed using the following indices: observed OTUs (reflecting species richness), the Chao1 index (estimating total community species richness), the Shannon index (integrating both species richness and evenness), and the Pielou’s evenness index (reflecting the uniformity of species distribution). β-diversity was analyzed to examine differences in microbial community structure using Bray–Curtis distance-based principal coordinate analysis (PCoA). Statistical significance of group differences was tested using PERMANOVA (Adonis) with 999 permutations. All analyses of bacterial α-diversity number β-diversity data was carried out in QIIME2. The relationship between microbial data and environmental factors was analyzed using redundancy analysis (RDA) in Canoco 5, with graphical representations created using Origin 2024.

## 3 Results

### 3.1 Effects of combined application of two biofertilizers and P fertilizer on soil nutrients

The application of chemical fertilizer (TSP) at different rates, combined with two biofertilizers (BF1 and BF2) increased soil SOM and TK, though the changes were not significant. The application of BF1 with varying phosphorus levels had no significant effect on soil TN and TP. However, under BF2 combined with P100, both TN and TP in the soil were significantly increased. In contrast, under the reduced phosphorus treatments (P85 and P70), although there was an increase in TN and TP, the changes were not significant. After applying BF1 with P100 and P85, both AP and AK in the soil were significantly enhanced (*p* < 0.05), with no significant difference observed between P100 and P85 in terms of soil AP and AK.

### 3.2 Effects of combined application of two biofertilizers and phosphorus fertilizer on soil bacterial communities

After the application of P100 combined with BF1, both the OTU and Chao1 indices of soil bacteria significantly increased, while after combining BF2, only the Chao1 index showed a significant increase (*p* < 0.05). Shannon-Wiener index of soil bacteria significantly increased after P100 combined with BF1, but both the Shannon-Wiener and Pielou indices significantly decreased after the application of BF2 (*p* < 0.05). No significant differences in the Shannon-Wiener and Pielou indices of soil bacteria were observed between the P85 and P70 treatments combined with BF2 and the CK treatment. At the phylum level, the application of BF2 combined with different phosphorus fertilizer treatments had a more significant impact on the soil bacterial community structure compared to BF1 (Figure 3).

Compared to the CK treatment, the application of BF1 significantly increased the relative abundance of *Acidobacteriota* at the phylum level, while the relative abundance of *Gemmatimonadota* and *Bacteroidota* significantly decreased (*p* < 0.05; Figure 4a). Following BF2 application, only the relative abundance of *Gemmatimonadota* significantly decreased (*p* < 0.05). However, under phosphorus reduction treatments, particularly the P85 treatment, the relative abundance of *Pseudomonadota* and *Gemmatimonadota* significantly decreased, while *Acidobacteriota* and *Planctomycetota* significantly increased in the community (*p* < 0.05; Figures 4a, 5).

Compared to the CK treatment, the application of BF1 significantly increased the relative abundance of Anaerobic and Biofilm-forming functional bacteria in the soil community (Figure 4b). Following BF2 application, the relative abundance of Pathogenic functional bacteria in the soil significantly increased. However, after phosphorus reduction, especially under the P85 treatment, there were no significant differences in the relative abundance of Pathogenic and Stress-tolerant functional bacteria compared to the CK treatment, while the relative abundance of Biofilm-forming functional bacteria significantly increased (Figure 4b).

Redundancy analysis results show that after the application of BF1 combined with different phosphorus treatments (Figure 5), *Acidobacteriota*, *Chloroflexota*, and *Actinomycetota* were the main factors influencing soil TP and TN. In contrast, after the application of BF2 combined with different phosphorus treatments, *Patescibacteria* and *Planctomycetota* were the primary factors affecting soil SOM, TN, and AK.

## 4 Discussion

### 4.1 Effects of phosphorus reduction combined with two biofertilizers on soil nutrients

**FIGURE 1:**
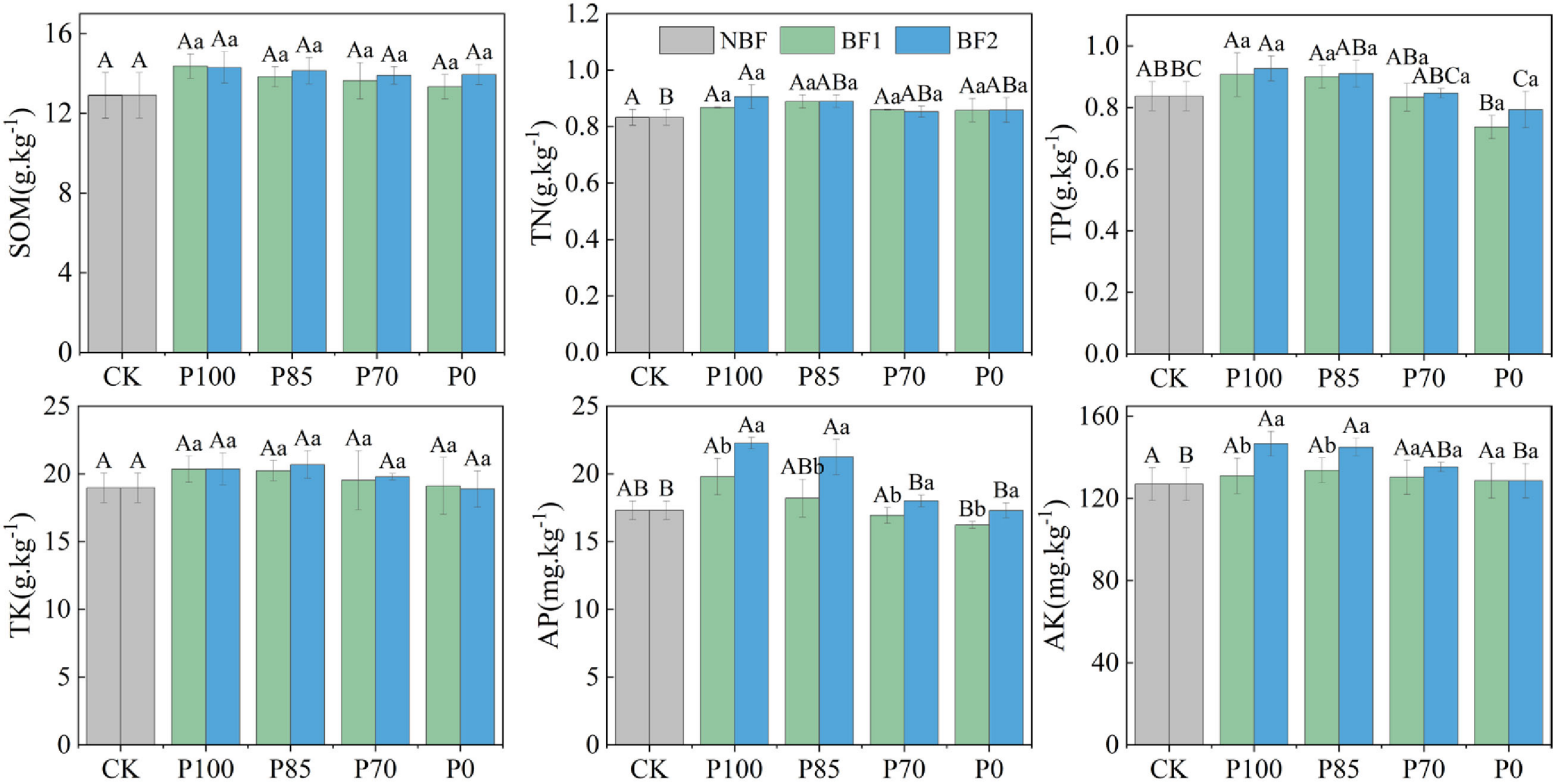
Effects of combined application of two biofertilizers and phosphorus fertilizer on soil organic matter (SOM), total nitrogen (TN), total phosphorus (TP), total potassium (TK), available phosphorus (AP), and available potassium (AK). Different uppercase letters in the figure indicate significant differences among different phosphorus fertilizer treatments under the same microbial fertilizer treatment, while lowercase letters denote significant differences among various microbial fertilizer treatments under the same phosphorus fertilizer application. BF1 and BF2 indicate *Bacillus subtilis*-based and *Bacillus mucilaginosus*-based biofertilizers, respectively.

Phosphorus is a key nutrient for crop growth and development; however, excessive application can exacerbate soil environmental stress and lead to non-point source pollution (Bindraban et al., 2020). A moderate reduction in phosphorus fertilization, combined with biofertilizers, can improve soil microbial communities and enhance the soil’s self-regulation capacity (Liu et al., 2022). Studies have shown that through scientifically managed phosphorus reduction and biofertilizer application, the levels of total phosphorus (TP) and available phosphorus (AP) in the soil can be maintained at optimal levels, while crop yields remain stable (Withers et al., 2017; Li Z. et al., 2022; Mącik et al., 2022; Liu et al., 2022). This finding is supported by our results, where no significant differences in TP, AP, and available potassium (AK) were observed between the P85 (15% reduced phosphorus with BF2) and P100 (standard phosphorus with BF2) treatments (Figure 1). This can be attributed to two main reasons: (1). *B. mucilaginosus* (BF2) assimilates some phosphorus during growth to form microbial biomass phosphorus, which, upon the bacteria’s death or dormancy, can be mineralized and released back into the soil, enhancing available phosphorus content. (2). The colonization of *B. mucilaginosus* enhances the phosphate-solubilizing action of organic acids and protons secreted by plant roots, forming a root-bacteria synergistic effect that further promotes phosphorus availability (Basak and Biswas, 2009). This indicates that reducing phosphorus fertilization by 15% combined with biofertilizer application does not decrease phosphorus availability in the soil. Furthermore, the combination of BF2 led to an increase in AK content, likely due to the ability of *B. mucilaginosus* to secrete organic acids (such as citric and oxalic acids) and exopolysaccharides that dissolve poorly soluble potassium minerals (e.g., potassium feldspar, mica), converting fixed potassium (K) into a form that plants can absorb, thus enhancing soil’s available potassium content (Basak and Biswas, 2009; Mącik et al., 2022). Additionally, although soil nutrients such as soil organic carbon (SOC), total nitrogen (TN), and TP slightly increased after the application of BF1 with phosphorus reduction treatments, the differences were not significant. This may be attributed to the fact that BF1’s functions are more inclined toward suppressing soilborne pathogens and promoting plant growth.

### 4.2 Effects of phosphorus reduction combined with two biofertilizers on soil bacteria

Soil microorganisms are the core driving force behind maintaining soil ecosystem functions. Changes in microbial community structure directly impact key processes such as soil nutrient cycling, organic matter stability, and pollutant degradation, thereby influencing soil health and productivity (Schmidt et al., 2019; Prasad et al., 2021). Our results show that after the application of BF1, the OTU, Chao1, and Shannon-Wiener indices of soil bacteria significantly increased. This is likely due to *B. subtilis* (BF1) breaking down organic matter to release nutrients, suppressing pathogenic bacteria to reduce competitive pressure, and optimizing the microenvironment, which synergistically drives increased bacterial diversity and abundance (Kovács, 2019; Liu et al., 2021).

**FIGURE 2:**
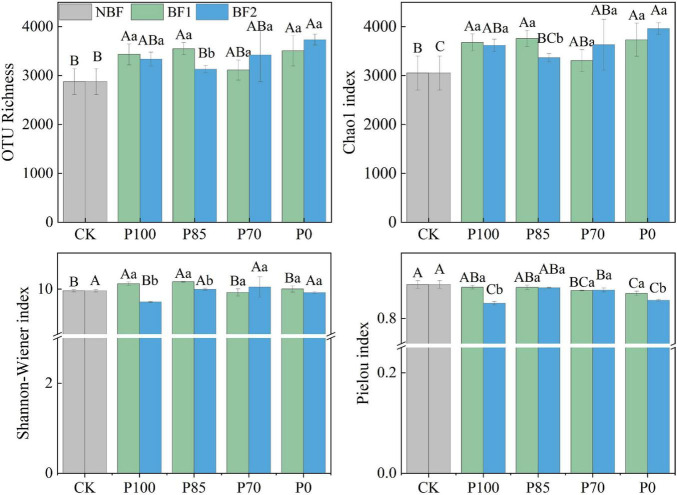
Effects of combined application of two biofertilizers and phosphorus fertilizer on soil bacterial diversity. Different uppercase letters in the figure indicate significant differences among different phosphorus fertilizer treatments under the same microbial fertilizer treatment, while lowercase letters denote significant differences among various microbial fertilizer treatments under the same phosphorus fertilizer application. BF1 and BF2 indicate *Bacillus subtilis*-based and *Bacillus mucilaginosus*-based biofertilizers, respectively.

**FIGURE 3:**
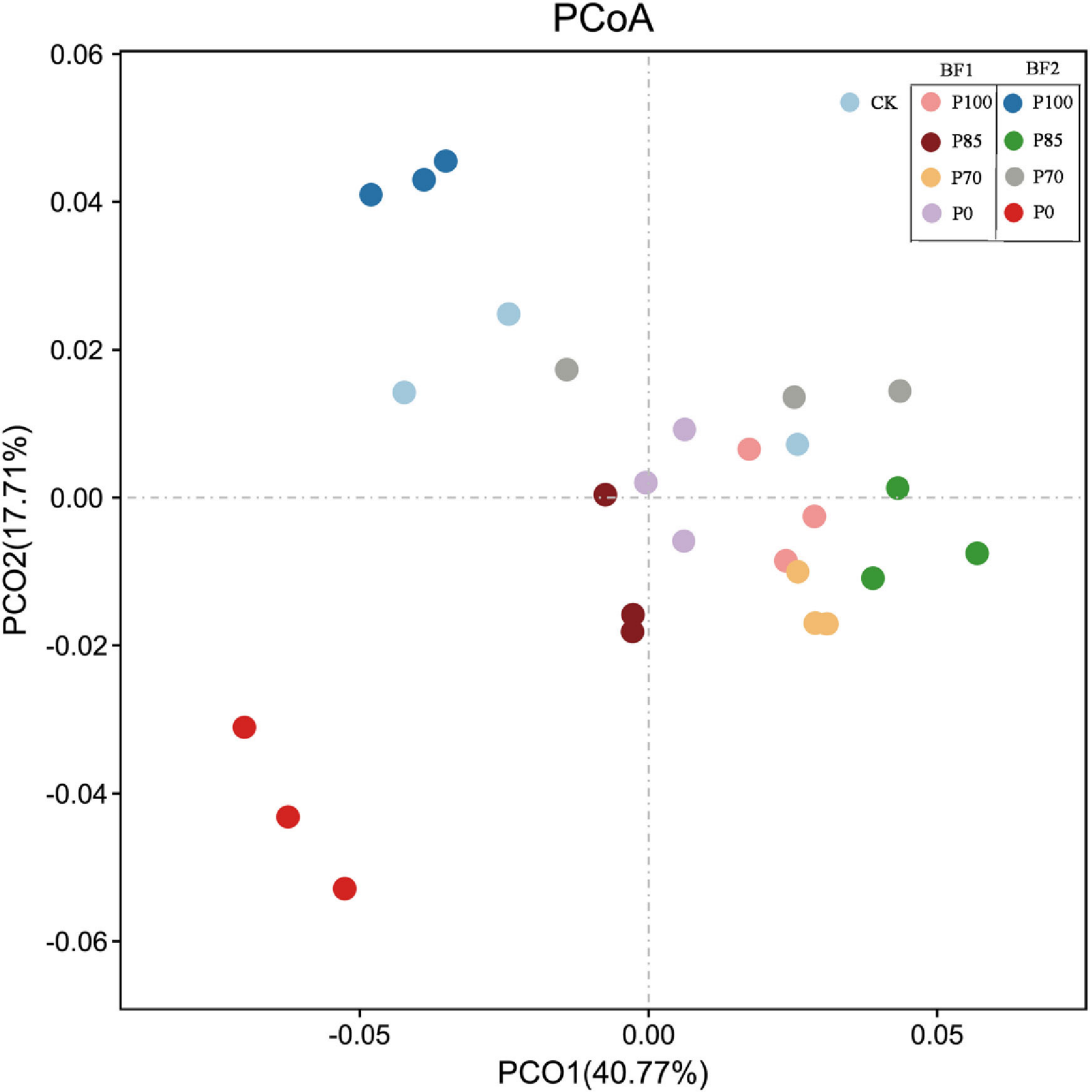
Effects of combined application of two biofertilizers and phosphorus fertilizer on bacterial community structure. BF1 and BF2 indicate *Bacillus subtilis*-based and *Bacillus mucilaginosus*-based biofertilizers, respectively.

In contrast, after the application of BF2, no significant differences in OTU or Chao1 indices were observed, but the Shannon-Wiener and Pielou indices of soil bacteria significantly decreased (Figure 2). This could be because *B. mucilaginosus* (BF2) selectively releases mineral nutrients and metabolic products, enriching oligotrophic functional bacterial communities, thereby intensifying interspecies resource competition. This results in a higher dominance of certain species and lower evenness in the soil microbial community, maintaining stability in species richness (OTU, Chao1) but significantly reducing diversity indices (Shannon/Pielou) (Liu et al., 2021). Moreover, community structure analysis of soil bacteria also indicates that BF2 application leads to more significant changes in bacterial community structure compared to BF1 (Figure 3).

After reducing phosphorus fertilization by 15%, bacterial OUT, Chao1 indices and Shannon-Wiener index under the BF2 treatment did not show significant changes (*p* > 0.05), but under the BF1 treatment, the diversity index significantly increased (Figure 2, *p* < 0.05). This increase in diversity under low-phosphorus conditions is likely due to *B. mucilaginosus* solubilizing and releasing phosphorus from insoluble compounds, which enhances microbial niche differentiation and functional redundancy, while phosphorus addition reduces resource competition and cooperative interaction demands (Kovács, 2019; Liu et al., 2022). Furthermore, under the P70 treatment, where phosphorus was reduced by 30%, there were decreases in total nitrogen, total phosphorus, available phosphorus, and available potassium in the soil (Figure 1), which could negatively affect crop yield, this may be due to phosphorus fertilizer reduction directly decreases the soil available phosphorus pool. As phosphorus serves as both an energy carrier and a critical component of nucleic acids, its deficiency may inhibit microbial organic nitrogen mineralization capacity, consequently leading to reduced soil total nitrogen accumulation (Liu et al., 2022). Furthermore, the synergistic phosphorus-potassium co-uptake effect becomes weakened under these conditions (Mącik et al., 2022; Liu et al., 2022). These results suggest that a 15% reduction in phosphorus fertilization (P85) combined with biofertilizer application does not negatively affect soil bacterial diversity or nutrient availability, and the biofertilizer facilitates nutrient cycling (Billah et al., 2019).

After the application of biofertilizers, functional microorganisms drive the rapid turnover of dominant species through mechanisms such as niche competition (where metabolites preempt resources) and allelopathic inhibition (targeted antagonism of competing microbial communities) (Kovács, 2019; Liu et al., 2022). Therefore, functional microorganisms play a direct and leading role in community construction. Our results indicate that after the application of BF1 (*Bacillus subtilis*), the relative abundance of *Acidobacteriota* at the phylum level significantly increased, while *Gemmatimonadota* and *Bacteroidota* significantly decreased. This phenomenon can be attributed to *B. subtilis*-mediated soil acidification through organic acid secretion and carbon source availability modification, which selectively enriches acidophilic *Acidobacteriota* while suppressing neutrophilic *Gemmatimonadota* and *Bacteroidota* populations, ultimately leading to microbial community restructuring (Yongling Hongjuan Zhang et al., 2024).

**FIGURE 4:**
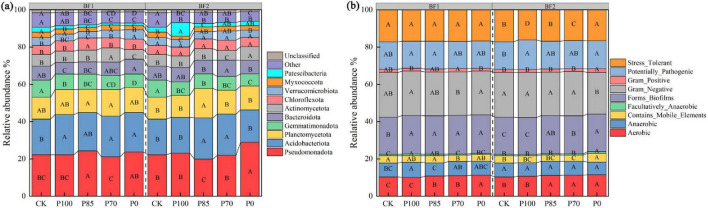
Effects of combined application of two biofertilizers and phosphorus fertilizer on bacterial community composition **(a)** and phenotypic classification **(b)**. Different uppercase letters in the figure indicate significant differences among different phosphorus fertilizer treatments under the same microbial fertilizer treatment. BF1 and BF2 indicate *Bacillus subtilis*-based and *Bacillus mucilaginosus*-based biofertilizers, respectively.

**FIGURE 5:**
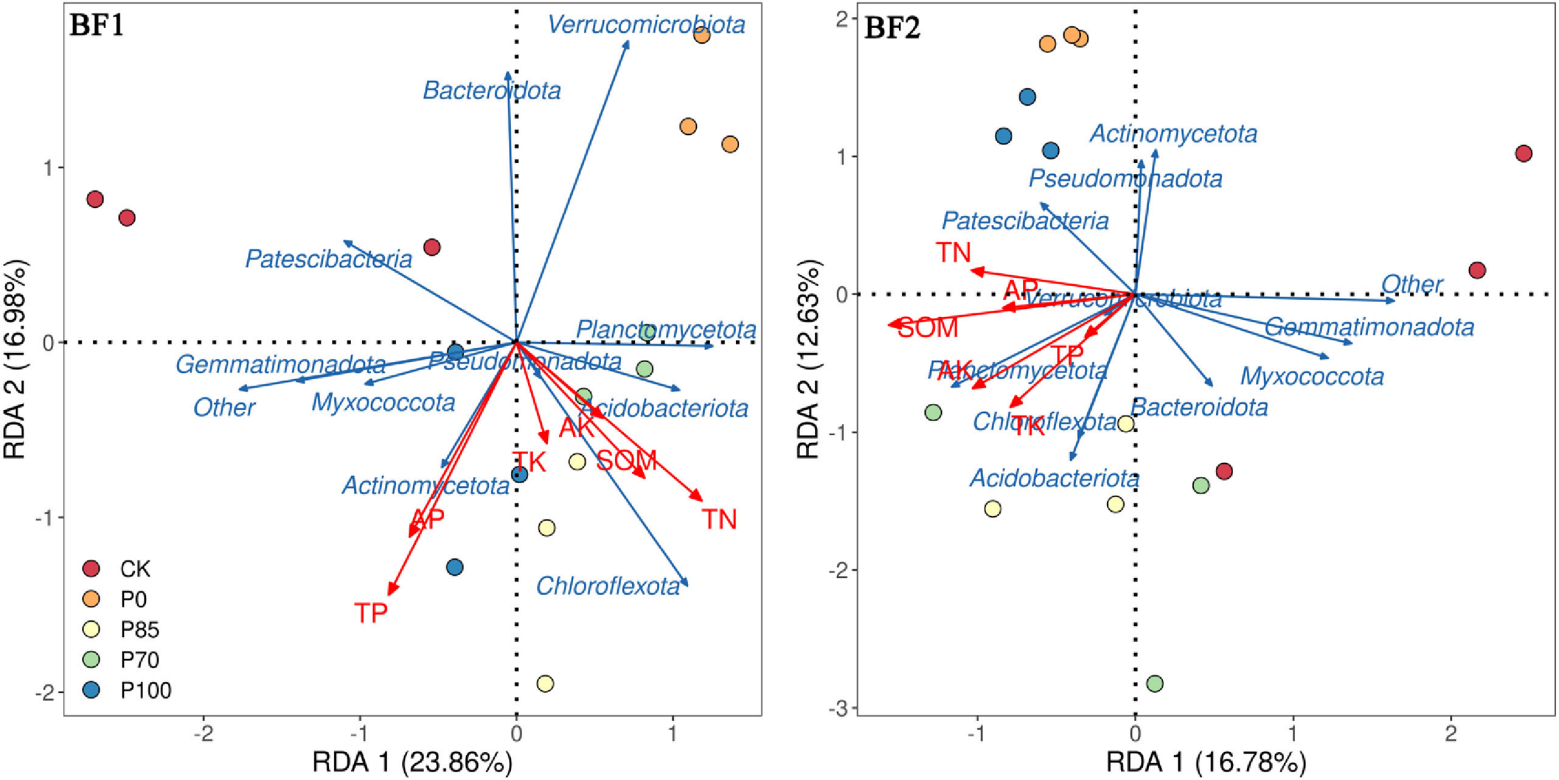
The relationship between bacterial community structure and soil nutrients following the application of biofertilizers combined with phosphorus fertilizers. BF1 and BF2 indicate *Bacillus subtilis*-based and *Bacillus mucilaginosus*-based biofertilizers, respectively.

Following the application of BF2 (*B. mucilaginosus*), only the relative abundance of *Gemmatimonadota* decreased significantly, likely due to the production of organic acids during the metabolic processes of *B. mucilaginosus*, which lowers the soil pH. This acidic environment may inhibit the growth of *Bacteroidota*, thus reducing their proportion in the community (Sun et al., 2015). However, under the phosphorus reduction treatments (e.g., P85), the relative abundance of *Pseudomonadota* and *Gemmatimonadota* in the microbial community significantly decreased, while *Acidobacteriota* and *Planctomycetota* increased significantly (Figures 4a, 5). This is likely because phosphorus is a key element for microbial growth, and phosphorus reduction may limit the growth of microorganisms such as *Pseudomonadota* and *Bacteroidota*, which require sufficient phosphorus for growth, leading to a decrease in their abundance (Glick, 2012).

After the application of BF1 (*B. subtilis*), the relative abundance of anaerobic bacteria and biofilm-forming bacteria significantly increased. This may be due to the metabolic activity of *B. subtilis* in the soil, which breaks down organic matter and releases nutrients that other microorganisms can utilize. These organic materials provide carbon sources for anaerobic bacteria, promoting their growth (Kovács, 2019). Following the application of BF2 (*B. mucilaginosus*), the relative abundance of pathogenic bacteria significantly increased, while the abundance of stress-tolerant bacteria remained largely unchanged. Additionally, biofilm-forming bacteria significantly increased in abundance (Basak and Biswas, 2009). The application of BF2 significantly increased the relative abundance of pathogenic bacteria and biofilm-forming bacteria, while showing no notable effect on stress-tolerant bacteria. This phenomenon may be attributed to: (1) nutrient release through BF2-mediated solubilization promoting pathogenic bacterial proliferation; (2) potential suppression of pathogen-antagonistic microorganisms by BF2 metabolites; (3) provision of extracellular polysaccharides (EPS) as a matrix for biofilm formation. The stability of stress-tolerant bacteria could result from unaltered soil stress conditions or functional redundancy within microbial communities (Basak and Biswas, 2009; Li Z. et al., 2022).

Under phosphorus reduction treatments, such as P85, there were no significant changes in the relative abundance of pathogenic and stress-tolerant functional bacteria, but the abundance of biofilm-forming functional bacteria increased significantly. This suggests that moderate phosphorus reduction benefits the growth of beneficial bacterial functional groups and improves soil fertility and plant health (Liu et al., 2022; Mącik et al., 2022).

Bacteria influence soil nutrient transformation, cycling, and availability through various mechanisms, thereby improving the soil nutrient status (Gao et al., 2022; Liu et al., 2022). RDA (Redundancy Analysis) results indicate that after the application of BF1 combined with different phosphorus fertilizers, *Acidobacteriota*, *Chloroflexota*, and *Actinomycetota* were the primary factors influencing soil total phosphorus (TP) and total nitrogen (TN). In contrast, after the application of BF2 with different phosphorus fertilizers, *Patescibacteria* and *Planctomycetota* were the major factors affecting soil soil organic matter (SOM), TN, and available potassium (AK) (Figure 5). This suggests that different biofertilizers, by modulating microbial communities, can optimize soil nutrient cycling and enhance soil quality (Liu et al., 2022).

## 5 Conclusion

Our findings demonstrate that a 15% phosphorus reduction combined with biofertilizer application (BF1/BF2) effectively improves soil nutrient status while differentially altering bacterial communities: BF1 significantly enhanced microbial diversity and *Pseudomonadota* (carbon cycle), whereas BF2 markedly increased available phosphorus/potassium and enriched *Acidobacteriota*/*Planctomycetota* (carbon-nitrogen cycling), with both biofertilizers promoting aerobic and biofilm-forming bacteria. RDA revealed *Chloroflexota* (nitrogen cycling) as the primary driver of nutrient dynamics under BF1, while BF2 effects were mediated by coordinated action of *Acidobacteriota*, *Chloroflexota*, and *Actinomycetota* (organic matter degradation). This optimized phosphorus-reduction strategy with biofertilizers enhances nutrient availability, improves microbial diversity, and represents a sustainable approach for maintaining soil health and agricultural productivity.

## Data Availability

The raw sequencing data supporting the findings of this study have been deposited in the NCBI Sequence Read Archive (SRA) under the BioProject accession number PRJNA1330030.
