# Correction: Impact of phosphorus reduction combined with biofertilizer application on soil nutrients and microbial communities in arid oasis agricultural areas

**DOI:** 10.3389/fmicb.2025.1724784

**Published:** 2025-10-27

**Authors:** Frontiers Production Office

**Affiliations:** Frontiers Media SA, Lausanne, Switzerland

**Keywords:** phosphorus reduction, biofertilizers, soil available nutrients, microbial diversity, carbon-nitrogen cycling, sustainable agriculture

Authors Yong Ling Zhang, Yun Chen Zhao, Tongfen Li, Hong Yu Cheng, and Hong Juan Zhang were erroneously spelled as Lin Yong Zhang, Cheng Yun Zhao, Tengfei Li, Yu Hon Cheng and Juan Hon Zhang.

The order of authors in the author list of the published paper was erroneously given as:

Rang Xiao, Juan Hon Zhang, Yu Hon Cheng, Tengfei Li, Cheng Yun Zhao and Lin Yong Zhang.

The correct author list reads:

Yong Ling Zhang, Rang Xiao, Yun Chen Zhao, Tongfen Li, Hong Yu Cheng, and Hong Juan Zhang

The original version of this article has been updated.

